# A Thermodynamic Perspective on Potential G‐Quadruplex Structures as Silencer Elements in the MYC Promoter

**DOI:** 10.1002/chem.202002985

**Published:** 2020-11-19

**Authors:** Jagannath Jana, Klaus Weisz

**Affiliations:** ^1^ Institute of Biochemistry Universität Greifswald Felix-Hausdorff-Str. 4 17487 Greifswald Germany

**Keywords:** calorimetry, c-myc, G-quadruplexes, thermodynamics

## Abstract

Multiple G‐tracts within the promoter region of the *c‐myc* oncogene may fold into various G‐quadruplexes with the recruitment of different tracts and guanosine residues for the G‐core assembly. Thermodynamic profiles for the folding of wild‐type and representative truncated as well as mutated sequences were extracted by comprehensive DSC experiments. The unique G‐quadruplex involving consecutive G‐tracts II–V with formation of two one‐nucleotide and one central two‐nucleotide propeller loop, previously proposed to be the biologically most relevant species, was found to be the most stable fold in terms of its Gibbs free energy of formation at ambient temperatures. Its stability derives from its short propeller loops but also from the favorable type of loop residues. Whereas quadruplex folds with long propeller loops are significantly disfavored, a snap‐back loop structure formed by incorporating a 3’‐terminal guanosine into the empty position of a tetrad seems highly competitive based on its thermodynamic stability. However, its destabilization by extending the 3’‐terminus questions the significance of such a species under in vivo conditions.

## Graphical abstract

**Multiple guanosine tracts** within the promoter region of the *c‐myc* oncogene may fold into various G‐quadruplex structures to act as gene regulatory elements. A thermodynamic profiling of quadruplex folding identifies most stable topologies at ambient temperatures.

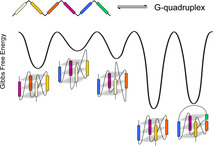

## Introduction

Guanine(G)‐rich sequences have the propensity to fold into non‐canonical tetra‐stranded structures called G‐quadruplexes (G4s). These are formed by stacking of planar hydrogen‐bonded guanine quartets in the presence of cations like Na^+^ or K^+^ that stabilize the stacked quartet arrangement by their coordination within the central cavity of the G‐core. Due to an overrepresentation of potential quadruplex‐forming sequences in promoter regions of various proto‐oncogenes like *c‐myc*, *c‐kit*, or *Bcl‐2*, G4 structures within a cellular environment are considered attractive targets for novel therapeutics. Thus, the *c‐myc* oncogene codes for a protein that may act as both transcriptional activator and repressor, being involved in the regulation of various genes linked to proliferation and growth arrest.^[1]^ Because overexpression of *c‐myc* was found to be associated with a wide range of human cancers,[^2^, ^3^] its complex transcriptional regulation employing multiple promoters has been the subject of intense research over the past three decades. As a result, the so‐called nuclease hypersensitivity element III_1_ (NHE III_1_), a 27 base pair sequence located upstream of the P1 promoter, was identified as a major control element of *c‐myc* expression.[^4^, ^5^] Notably, double‐stranded NHE III_1_ was found to be in equilibrium with a non‐canonical quadruplex formed after duplex unwinding by intrastrand folding of its purine‐rich noncoding strand.^[6]^ Two different G‐quadruplex structures for the single‐stranded NHE III_1_ were identified and characterized based on DMS footprinting experiments.^[7]^ Following initial assignments to an antiparallel basket and a chair topology, compelling evidence of their folding into three‐layered parallel quadruplexes with three propeller loops came from subsequent NMR studies on truncated and mutated sequences.^[8]^

**Figure 1:**
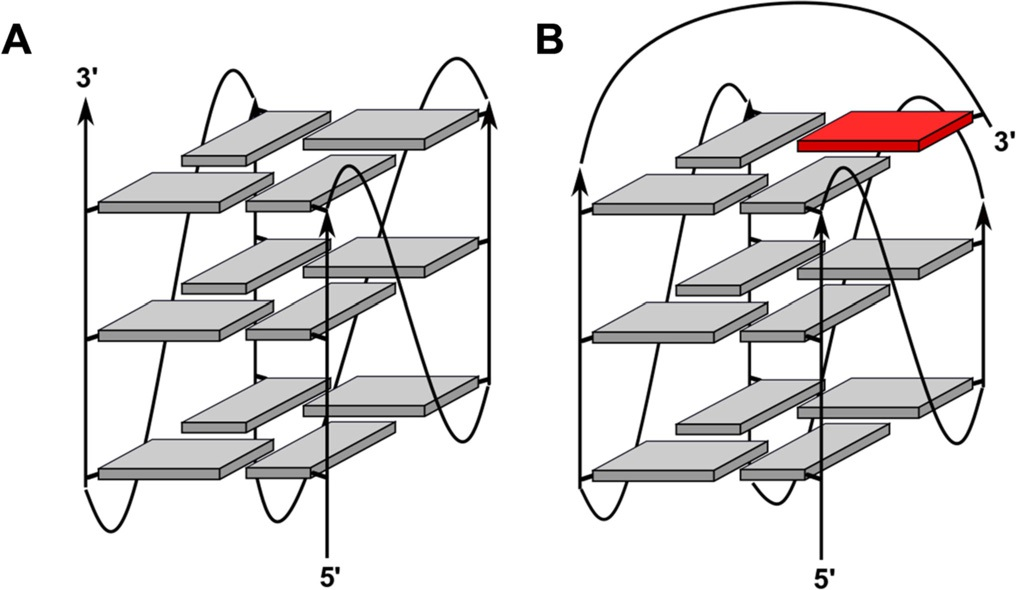
Schematic representation of a parallel (A) and fold‐back quadruplex (B); grey and red rectangles denote G residues in *anti* and *syn* conformation, respectively.

With its six guanine tracts composed of two to four consecutive G residues, the wild‐type 27mer *MYC* single strand may fold into multiple G‐quadruplex structures with various loop architectures depending on the set of G‐tracts recruited to form the G‐quadruplex core (Table 1). Site‐directed mutagenesis together with polymerase stop and luciferase reporter assays in the absence and presence of the G‐quadruplex binding porphyrin TMPyP4 pointed to G‐tracts II–V as being involved in a biologically relevant G4 repressor element whose formation decreases *c‐myc* expression at the RNA as well as the protein level.[^7^, ^9^] Because the first G‐tract was suggested to not be engaged in the transcriptional gene regulation, subsequent structural studies mostly focused on truncated sequences only involving the four central G‐tracts of *MYC* as models of the physiologically active G‐quadruplex (Figure 1 A).^[10]^ As a consequence of two stretches comprising four guanines, their folding into a parallel three‐layered G‐quadruplex allows for the formation of four different loop isomers.[^11^, ^12^] However, a quadruplex with two one‐nucleotide (1‐nt) and a central 2‐nt propeller loop was suggested to predominate in buffer solution.^[8]^ Even adding more diversity, expansion of the truncated sequence to also include the 3’‐terminal G_2_‐tract VI of the wild‐type *MYC* resulted in a different major G‐quadruplex species that features a snap‐back loop and allows the 3’‐terminal guanine base to fill an empty guanine position within the 3’‐tetrad (Figure 1 B).^[13]^

**Table 1:** Sequence for the wild‐type *MYC* G‐rich strand of NHE III_1_ and for sequence variants. G‐tracts I–VI are underlined and G substitutions are marked in italics.

Name	Sequence	
*MYC*	TGGGGAGGGTGGGGAGGGTGGGGAAGG	27mer
*MYC*‐Δ5,6	*T*TGGGGAGGGTGGGGAGGGT*T*	21mer
*MYC*‐Δ3,6	*T*TGGGGAGGGT*TTTA*AGGGTGGGGAA*T*	27mer
*MYC*‐Δ2,6	*T*TGGGGA*TTT*T*A* GGGAGGGTGGGGAA*T*	27mer
*MYC*‐Δ1,6	*T*GAGGGTGGGGAGGGTGGGGAA	22mer
*MYC_TT_*‐Δ1,6	*TT* GGGTGGGGAGGGTGGGG *TT*	21mer
*MYC*‐Δ1,6[1.2.1]	*T*GAGGGTGGG *T*AGGGTGGG *T*AA	22mer
*MYC*‐Δ1,6[2.1.2]	*T*GAGGGT*T* GGGAGGGT*T* GGGAA	22mer
*MYC*‐Δ1[1.3.1]	*T*GAGGGTGG *T*GAGGGTGGGGAAGG	24mer
*MYC_del_*‐Δ1[1.1.1]	*T*GAGGGTGG *T* GGGTGGGGAAGG	22mer
*MYC*‐Δ1[1.3.1]T	*T*GAGGGTGG *T*GAGGGTGGGGAAGGT	25mer
*MYC*‐Δ1[1.3.1]TT	*T*GAGGGTGG *T*GAGGGTGGGGAAGGTT	26mer

Due to the importance of the G‐quadruplex structures formed by NHE‐III_1_ for understanding *c‐myc* expression and for a rational design of drugs targeting the *MYC* sequence, various efforts have been directed towards identifying and characterizing the physiologically most relevant quadruplex species.[^11^, ^14^] However, major quadruplexes formed under in vivo conditions will not only depend on their intrinsic thermodynamic stability but also on their folding kinetics and on the differential binding of multiple transcription factors like NM23‐H2 or nucleolin.[^15^, ^16^] Such a complex situation requires an individual assessment of binding processes, G4 folding kinetics as well as G4 thermodynamic stability. Previous studies have evaluated G4 thermodynamics within a controlled in vitro environment by the determination of melting temperatures and also by van ’t Hoff analyses based on a two‐state transition, yet rigorous and systematic investigations on *MYC*‐derived sequences are largely missing. We here report on a detailed thermodynamic profiling of an extensive set of putative *MYC* quadruplex folds by microcalorimetric methods to allow for a model‐free extraction of thermodynamic parameters. The present studies not only focus on specific loop isomers of a single fold but also on G‐quadruplex structures with the recruitment of different sets of G‐stretches. Although insufficient to fully describe G4 populations within the cellular environment, such a comprehensive thermodynamic profiling will add to our understanding of major G4 structures formed in vivo. On the other hand, knowing the folding thermodynamics of individual *MYC* sequences in detail gives valuable information on the impact of loop sequence, loop positioning, and loop length on the G4 stability of parallel quadruplexes in general.

## Results and Discussion

### NHE III_1_ sequence variants

For a detailed evaluation of quadruplex stabilities, various G‐rich sequences derived from the purine‐rich 27mer NHE III_1_ single strand termed *MYC* were employed (Table 1). Excluding its GG 3’‐terminus, the *MYC* sequence features five G‐tracts with potential folding into five different G4 species depending on the G‐tracts forming the G‐quadruplex core. In addition, one of the guanines at either the 5’‐ or 3’‐end of a G_4_‐tract is excluded from a G‐column in a three‐layered quadruplex. Given three G_4_‐tracts in *MYC*, this gives rise to 2^2^ or 2^3^ loop isomers for the five quadruplexes depending on their folding with participation of two or all three *MYC* G_4_‐tracts, respectively. Disregarding the terminal G_2_‐tract VI and assuming exclusive formation of all‐parallel G‐quadruplexes it can easily be seen that there are 28 possible species of three‐layered G4s.

Introducing mutations within G tracts constitutes a convenient strategy to block formation of otherwise competing G4 structures. Thus, selective G→T/A substitutions may restrict folding into quadruplexes with only one defined set of G‐columns or even provide for exclusive folding into a single loop isomer. However, to serve as good mimics of the wild‐type fold, trapped mutants are required to not noticeably affect the structure and thermodynamic stability when compared to the corresponding wild‐type sequence. In fact, due to their position within loop regions base substitutions are suggested to only have a small impact on the G4 structure, but some blurring in stability should be considered when discussing sequences structurally trapped through mutations and truncations (see below).[^17^, ^18^, ^19^]

The selection of truncated and/or substituted sequences is based on representatives that cover a wide range of structural diversity to provide for extensive information on structure‐stability relationships. For the sake of simplicity and consistency, sequences are named according to the G‐tracts not involved in tetrad formation (i.e., tract I and VI in *MYC*‐Δ1,6) and in keeping with the number of nucleotides within the propeller loops (i.e., two 1‐nt and a central 2‐nt propeller loop in *MYC*‐Δ1,6[1.2.1]). Sequence variants of *MYC*, that is, *MYC*‐Δ1,6[1.2.1],^[10]^ *MYC*‐Δ1[1.3.1],^[13]^ and *MYC*‐Δ3,6^[20]^ previously characterized in detail by solution NMR have been preferred over related sequences. Both *MYC*‐Δ1,6[1.2.1] (PDB ID 1XAV) and *MYC*‐Δ3,6 (PDB ID 6NEB) fold into a single quadruplex of parallel topology (Figure 1 A) but differ in the length of their central loop, with *MYC*‐Δ3,6 exclusively forming a 1.6.1 loop isomer. On the other hand, by also recruiting the terminal G_2_‐tract and incorporating the 3’‐G residue into an empty position of the 3’‐tetrad, *MYC*‐Δ1[1.3.1] (PDB ID 2A5P) features an interrupted G‐column and a diagonal 4‐nt snap‐back loop bridging the 3’‐outer tetrad (Figure 1 B).

### Probing the G‐quadruplex topology by their CD signatures

**Figure 2:**
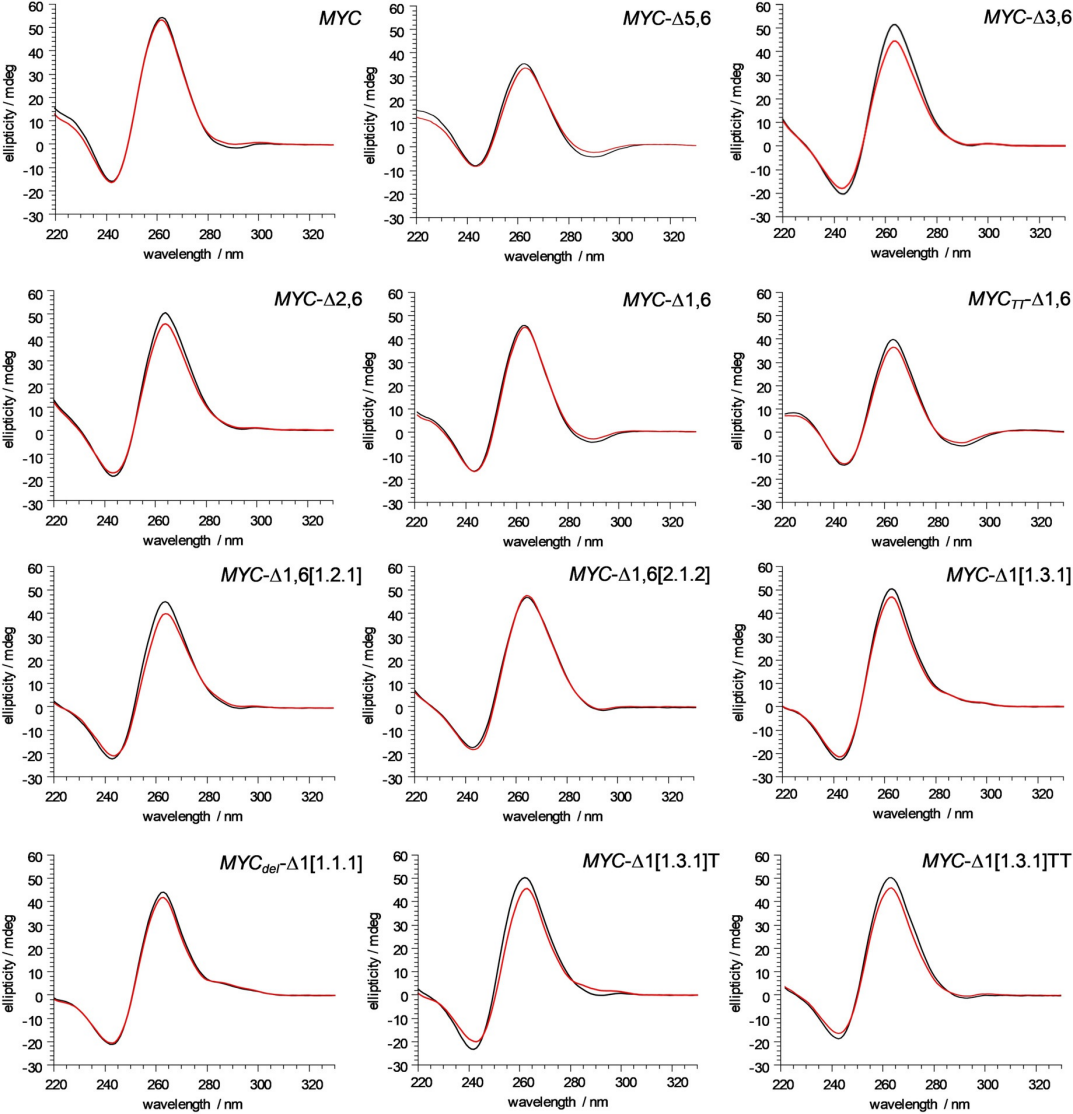
CD spectra of *MYC* sequence variants (5 μm) at 293 K in phosphate buffer, pH 7, with 10 mm (red) and 120 mm K^+^ (black).

For assessing quadruplex thermal stabilities and driving forces of G4 folding, differential scanning calorimetric measurements (DSC) were performed.^[21]^ In order to lower high melting temperatures for obtaining well‐defined post‐transitional baselines in the accessible temperature range as required for the extraction of reliable transition enthalpies, samples were dissolved in a low‐salt buffer with 10 mm potassium ions. Therefore, G4 folding was initially tested by a comparison of CD spectra acquired in both 10 mm and 120 mm K^+^ buffers (Figure 2). CD spectra of all sequences in potassium buffer feature negative and positive amplitudes at about 243 and 263 nm, typical of parallel quadruplexes with exclusive homopolar tetrad stacking. Whereas a broad low‐intensity shoulder between 280 and 300 nm distinguishes the snap‐back loop quadruplexes *MYC*‐Δ1[1.3.1] and *MYC_del_*‐Δ1[1.1.1] with the involvement of G‐tract VI from the regular parallel species with uninterrupted G‐tracts, a negative dip at 290 nm is noticeable in particular for *MYC*‐Δ5,6, *MYC*‐Δ1,6, and *MYC_TT_*‐Δ1,6. Importantly, except for minor variations in amplitude, CD signatures were conserved at the different salt concentrations with no apparent change in G4 topology.

### Probing the structural polymorphism of *MYC* variants

**Figure 3:**
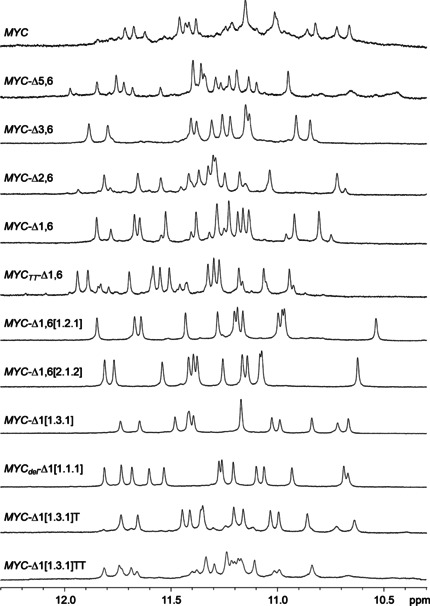
Imino proton NMR spectral region of *MYC* sequence variants (0.2 mm) at 298 K in 10 mm potassium phosphate buffer, pH 7.

^1^H NMR spectra of all *MYC*‐derived sequences were acquired to evaluate their folding and to test for structural heterogeneities under the solution conditions employed (Figures 3 and S1). Focusing on the Hoogsteen imino proton spectral region between 10.5 and 12.0 ppm, 12 resonances are generally expected for a single quadruplex with three G‐tetrad layers, corresponding to 3×4 hydrogen‐bonded guanine bases. Whereas the parent full‐length *MYC* sequence shows extensive polymorphism with multiple G4 species as suggested by its crowded imino proton spectral region, the spectra of some mutated sequences are in line with a single fold without noticeable additional species. These include not only *MYC*‐Δ1,6[1.2.1],^[10]^ *MYC*‐Δ1,6[2.1.2], *MYC*‐Δ1[1.3.1],^[13]^ and *MYC_del_*‐Δ1[1.1.1],^[22]^ but also *MYC*‐Δ3,6 with the formation of a single 1.6.1 loop isomer.^[20]^ Other sequences exhibit coexistence of different folds, however, a major species populated by ≥80 % predominates over a minor species in most cases.

A straightforward comparison and interpretation of thermodynamic parameters for G4 formation is affected by the coexistence of multiple topologies with equilibria possibly changed by outer conditions. However, other serious problems for any thermodynamic analysis may also result from sequences that partially form stable higher‐order G4 structures in vitro depending on sequence, ion concentration, but also on the particular annealing procedure, as has been reported recently.[^14^, ^23^, ^24^] Thus, the polymorphic *MYC* sequence was shown to have a strong tendency for intermolecular aggregation and with multimeric structures only melting at very high temperatures the formed fraction of higher‐order species may remain unnoticed in G4 denaturation studies. Although thermal stabilities as measured by melting temperatures remain unaffected, enthalpies and entropies of unfolding may consequently be too low and compromise an acceptable accuracy.

To assess the potential formation of multimeric structures we also performed non‐denaturing polyacrylamide gel electrophoresis for all sequences following annealing in a 10 mm potassium phosphate buffer used for NMR and DSC studies. Most of the sequences exhibit either a single or strongly predominating monomer band under the low‐salt conditions, differing in migration according to their number of residues (Figure S2). Yet, additional slower migrating bands for *MYC*‐Δ5,6 and in particular for *MYC*‐Δ1,6 and *MYC_TT_*‐Δ1,6 comprising about 30 % of the total in‐lane intensity hint at putative dimer formation for these mutant sequences. However, with no significant intensity of broadened signals in the ^1^H NMR spectra with their flat baseline (Figure 3), larger high‐melting G4 associates like G‐wire species can mostly be excluded for the latter sequences but may gain in relevance with buffers of higher ionic strength.

### Thermodynamic profiling of quadruplex formation by DSC

**Figure 4:**
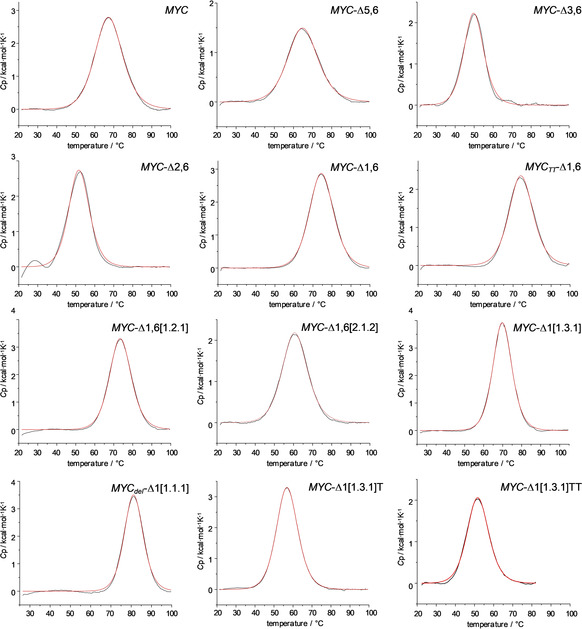
DSC thermograms for melting of *MYC* sequence variants in phosphate buffer, pH 7, in the presence of 10 mm K^+^. Fitted curves based on a non‐two‐state model with Δ*H* ^o^_cal_≠Δ*H* ^o^_vH_ are shown in red.

Representative examples of DSC melting profiles are shown in Figure 4. It should be noted that for some of the samples heating above 100 °C resulted in a gradual decrease of signal heights in subsequent heating cycles apparently due to partial heat‐induced damage of the DNA. Therefore, only those thermograms were used for evaluating the thermodynamics of folding that were reproducible with insignificant signal loss in a following scan.

All sequences feature monophasic transitions, albeit with different widths. Their symmetric profiles give no obvious indication of superimposed independent melting events. Of note, melting temperatures given by the maxima of the DSC curves agree within ±2 °C with previously reported values from CD melting experiments under the same buffer conditions for *MYC*‐Δ1,6[1.2.1] and *MYC_del_*‐Δ1[1.1.1]^[22]^ as well as from a separate CD‐based *T*_m_ determination for *MYC*‐Δ1,6[1.2.1] (data not shown). After proper baseline corrections, calorimetric enthalpies Δ*H* ^o^_cal_ of G4 folding were obtained by integration of the melting transition. Likewise, ensuring equilibrium conditions during thermal denaturation, molar entropies Δ*S* ^o^ at the melting temperature were calculated using standard thermodynamic relationships. These also enabled determination of the Gibbs free energy Δ*G* ^o^ at any reference temperature *T* from Δ*H* ^o^ and Δ*S* ^o^ when changes in molar heat capacities Δ*C*_p_^o^ are close to zero.[^21^, ^25^] In fact, although quadruplex folding is expected to be associated with a Δ*C*_p_^o^≠0, changes are generally too small to warrant their reliable extraction from the thermograms in case of the G4 transitions.^[19]^ Average values from three independent measurements on different samples are summarized in Table 2. Here, only model‐free calorimetric transition enthalpies Δ*H* ^o^_cal_ and entropies Δ*S* ^o^_cal_ are included because many of the sequences employed may fold into multiple quadruplex species. Consequently, observed ratios between calorimetric and van ’t Hoff enthalpies determined based on a two‐state approximation will hardly yield direct information on (un)folding intermediates and/or cooperative units.

**Table 2:** DSC‐derived thermodynamic parameters for G4 folding.^[a]^

Sequence	*T*_m_ [°C]^[b]^	Δ*H* ^o^_cal_ [kcal mol^−1^)^[b]^	Δ*S* ^o^_cal_(*T*_m_) [cal mol^−1^ K^−1^]^[c]^	Δ*G* ^o^_293_ [kcal mol^−1^]^[d]^	Δ*G* ^o^_310_ [kcal mol^−1^]^[d]^
*MYC*	67.7±0.5	−52.2±1.5	−153.3±4.4	−7.3±0.2	−4.7±0.2
*MYC*‐Δ5,6	65.3±0.2	−30.4±0.7	−89.7±2.1	−4.1±0.1	−2.5±0.1
*MYC*‐Δ3,6	49.9±0.0	−32.4±1.4	−100.2±4.3	−3.0±0.1	−1.3±0.1
*MYC*‐Δ3,6^[e]^	70.6±0.1	−40.6±2.1	−118.2±6.0	−6.0±0.3	−4.0±0.2
*MYC*‐Δ3,6^[f]^	41.7±0.3	−19.4±1.8	−61.5±5.9	−1.3±0.1	−0.3±0.1
*MYC*‐Δ2,6	51.8±0.1	−39.9±0.9	−122.9±2.9	−3.9±0.1	−1.8±0.1
*MYC*‐Δ1,6	74.7±0.2	−48.9±0.2	−140.6±0.4	−7.7±0.1	−5.3±0.1
*MYC_TT_*‐Δ1,6	74.4±0.1	−40.2±0.4	−115.7±1.0	−6.3±0.1	−4.3±0.1
*MYC*‐Δ1,6[1.2.1]	73.5±0.1	−50.5±0.6	−145.7±1.8	−7.8±0.1	−5.3±0.1
*MYC*‐Δ1,6[1.2.1]^[e]^	91.0±0.1	−65.5±1.6	−180.0±4.2	−12.7±0.3	−9.7±0.3
*MYC‐*Δ1,6[1.2.1]^[f]^	59.0±0.3	−37.9±3.2	−114.3±9.6	−4.4±0.3	−2.5±0.2
*MYC*‐Δ1,6[2.1.2]	60.7±0.2	−39.3±0.9	−117.8±2.9	−4.8±0.1	−2.8±0.1
*MYC*‐Δ1[1.3.1]	69.8±0.1	−51.2±1.0	−149.6±3.0	−7.4±0.2	−4.9±0.1
*MYC*‐Δ1[1.3.1]^[f]^	61.6±0.4	−40.2±2.5	−120.0±7.7	−5.0±0.3	−3.0±0.1
*MYC_del_*‐Δ1[1.1.1]	81.0±0.2	−42.5±1.8	−120.1±5.1	−7.3±0.3	−5.2±0.2
*MYC*‐Δ1[1.3.1]T	56.5±0.3	−49.3±1.9	−149.5±5.7	−5.5±0.2	−2.9±0.1
*MYC*‐Δ1[1.3.1]TT	51.9±0.1	−31.9±0.6	−98.2±1.8	−3.1±0.1	−1.5±0.1

[a] In 10 mm K^+^ phosphate. [b] Average value from three independent measurements. [c] Calculated at *T*_m_ from the relationship Δ*S* ^o^=Δ*H* ^o^_cal_/*T*_m_. [d] Calculated from the relationship Δ*G* ^o^=Δ*H* ^o^−*TΔS* ^o^ and Δ*C*_p_^o^=0. [e] In 120 mm K^+^ buffer. [f] In 120 mm Na^+^ buffer.

### Dependence on concentration and type of cations

Due to the high thermal stability of some quadruplexes and the need for a defined post‐transitional baseline for proper baseline corrections, G4 melting was shifted to lower temperatures by using a low‐salt buffer with 10 mm potassium phosphate, pH 7. To probe differential effects of cation concentration on thermodynamic profiles of the parallel quadruplexes, two representative G‐rich sequences, namely *MYC*‐Δ1,6[1.2.1] and *MYC*‐Δ3,6, each forming a well‐defined quadruplex species were also characterized in a high‐salt buffer with 120 mm potassium phosphate, pH 7 (Table 2, Figure S3). The significant rise in their thermal stability with a Δ*T*_m_ of about 20 °C can be attributed to a higher ionic strength of the buffer solution but also to the specific uptake of K^+^ ions upon G4 folding, being coordinated within the central G4 cavity as a prerequisite for quadruplex stabilization. From a thermodynamic perspective, an elevated potassium ion concentration results in a more exothermic folding of the parallel quadruplex, yet is accompanied by a higher entropic penalty. This points to stronger intramolecular interactions and a higher rigidity similar to observations reported for a DNA four‐way junction lacking more specific cation binding.^[26]^ Because the Δ*H°* contribution to the Gibbs free energy predominates even at higher temperatures, the overall thermodynamic stability of the quadruplexes will always increase in the accessible temperature range at higher K^+^ concentrations. On the other hand, relative stabilities among quadruplexes are anticipated to not be affected by changing K^+^ concentrations. It should be noted, however, that in addition to slower kinetics of (un)folding, absolute differences in enthalpies and Gibbs free energies between G4 species will generally show a gradual decrease with a lowered K^+^ concentration.^[17]^ Thus, ΔΔ*G*°_293_ between *MYC*‐Δ1,6[1.2.1] and *MYC*‐Δ3,6 amounts to −6.7 kcal mol^−1^ in 120 mm potassium phosphate buffer but only to −4.8 kcal mol^−1^ in 10 mm phosphate.

A better stabilization of guanine quadruplexes with K^+^ compared to Na^+^ ions was shown to almost equally depend on desolvation and the size of the alkali metal cation.^[27]^ To also test the impact of substituting potassium for sodium ions on the thermodynamic profile of G4 folding, additional DSC thermograms were acquired for *MYC*‐Δ1,6[1.2.1], *MYC*‐Δ1[1.3.1] and *MYC*‐Δ3,6. Apparently, replacing potassium by sodium ions destabilizes the parallel quadruplexes by lowering exothermicities of folding with only partial compensation through a reduced entropic penalty in close analogy to decreasing K^+^ concentrations. On the other hand, destabilization of parallel but selective stabilization of antiparallel conformations has been reported by Na^+^.^[28]^ Whereas both *MYC*‐Δ1,6[1.2.1] and *MYC*‐Δ1[1.3.1] maintain a parallel topology irrespective of metal cation, substituting potassium for sodium ions leads to (partial) refolding of the less stable *MYC*‐Δ3,6 to an antiparallel or (3+1) hybrid‐type G4 as apparent from the typical CD signature (Figure S4). Clearly, this prohibits a direct comparison of cation impact on the thermodynamic profile of the latter and sets limits to changes in solution conditions.

### G4 variants with different G‐tracts for G‐core assembly

To characterize the thermodynamics of G4 folding for the *MYC* sequence when recruiting a different set of G‐tracts, individual tracts were either truncated if located at the termini or blocked by G→T/A substitutions, forcing them to loop out between adjacent G‐columns. In the present study, G‐tracts I, II, III, or V were individually blocked in addition to truncating the *MYC* 3’‐terminus with its G_2_‐tract VI to give *MYC*‐Δ1,6, *MYC*‐Δ2,6, *MYC*‐Δ3,6, and *MYC*‐Δ5,6. Based on previous observations that addition of the two‐nucleotide GG 3’‐terminus will result in refolding of a *MYC*‐Δ1 parallel quadruplex into a quadruplex with an interrupted G‐tract and a snap‐back loop with positioning of the 3’‐terminal G into the empty position of the outer G‐core (Figure 1 B), folding of a sequence *MYC*‐Δ1(1.3.1) was also characterized in more detail. Because all of these *MYC*‐derived sequences carry G_4_ tracts, they are expected to fold into different loop isomers through register shifts.^[19]^ It should be noted, however, that a single G→A/T substitution within a central G‐tract of *MYC*‐Δ2,6 and *MYC*‐Δ1(1.3.1) was introduced for better comparison, reducing the number of available folding pathways.

Before discussing thermodynamic stabilities, it is instructive to look at the imino proton spectral region of the various sequences mimicking *MYC* G‐tract isomers. Notably, imino resonances for most folds suggest a rather homogenous G4 population with only small amounts of coexisting species (Figure 3). As reported previously, *MYC*‐Δ3,6 and *MYC*‐Δ1(1.3.1) form a single 1.6.1 parallel and a 1.3.1 snap‐back loop quadruplex, respectively. For *MYC*‐Δ1,6 and *MYC*‐Δ2,6 the major quadruplex coexists with some minor G4 species populated to ≈20 %. Inspection of the major imino resonances of parent *MYC*‐Δ1,6 and comparison with the previously assigned resonances of *MYC*‐Δ1,6[1.2.1] with its two additional G→T substitutions at the central intervening and 3’‐overhang sequences reveals their identical fold in line with corresponding thermodynamic stabilities and profiles (Table 2). Together with the favored fold of *MYC*‐Δ3,6 and *MYC*‐Δ1(1.3.1), it can thus be assumed that G_4_ tracts at 5’‐ and 3’‐termini strongly favor loop isomers that shift the excessive G to the overhang rather than to an internal propeller loop. Accordingly, the observed predominant G4 for *MYC*‐Δ2,6 likely constitutes the 6‐1‐1 loop isomer. On the other hand, *MYC*‐Δ5,6 exhibits a more significant heterogeneity with an imino proton spectral region apparently composed of at least two major quadruplex folds.

**Figure 5:**
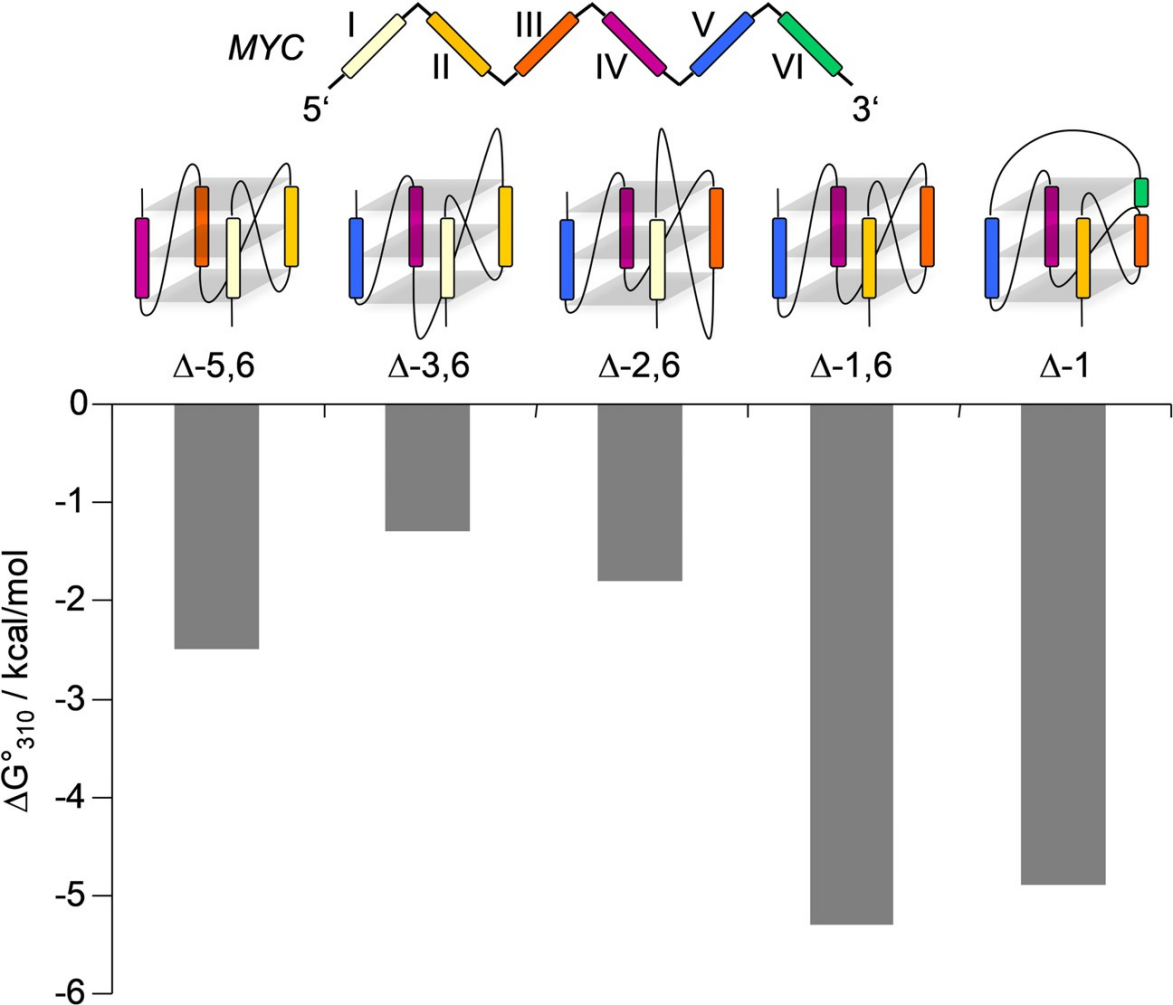
Gibbs free energy of quadruplex formation involving different G‐tracts for *MYC*‐derived sequences at 310 K. G‐tracts I–VI are color‐coded.

Gibbs free energies of the five quadruplexes differing in the identity of their G‐columns vary considerably by 4 kcal mol^−1^ at 310 K in a 10 mm K^+^ buffer, and differences tend to even increase at 293 K. *MYC*‐Δ1,6 and *MYC*‐Δ1[1.3.1] fold into the most stable G4 structures, not only being close in their Gibbs free energies but also in their enthalpic and entropic contributions. On the other hand, *MYC*‐Δ2,6 and especially *MYC*‐Δ3,6 with their single long loop reveal a rather low stability (Figure 5). Clearly, the latter two folds suffer from a long propeller loop known to generally compromise quadruplex stability. Whereas longer lateral loops have previously found to confer more exothermic heat to G4 folding through additional loop interactions and the uptake of counterions and water,^[29]^ this does not apply to the present propeller loops. Thus, both *MYC*‐Δ2,6 and *MYC*‐Δ3,6 exhibit a less negative molar transition enthalpy Δ*H* ^o^_cal_ but also a lowered entropic penalty upon folding when compared to the most stable *MYC*‐Δ1,6 and *MYC*‐Δ1[1.3.1]. Noticeably, quadruplexes derived from *MYC*‐Δ5,6 share a rather similar sequence with *MYC*‐Δ1,6 but have a significantly more positive Gibbs free energy by about 3 kcal mol^−1^ when compared to the latter (see below). Also, both favorable enthalpy and unfavorable entropy of folding are conspicuously smaller for *MYC*‐Δ5,6 that was suggested to not be involved in the biologically relevant *MYC* quadruplex.

### Snap‐back loop quadruplexes

Based on its thermodynamic stability and also suggested by previous NMR studies,^[13]^ the snap‐back loop quadruplex encompassing the 3’‐terminal GG‐tract may effectively compete with a regular parallel quadruplex in case of the full‐length *MYC* sequence. Consequently, such snap‐back loop quadruplexes have been targeted by various ligands with sometimes promising binding affinities.[^13^, ^22^, ^30^] To get more insight into the impact of additional modifications on the stability of the snap‐back loop structure, we shortened its two‐tetrad bridging central propeller loop by deletion of two nucleotides to give *MYC_del_*‐Δ1[1.1.1] or added one and two additional 3’‐terminal T residues in *MYC*‐Δ1[1.3.1]T and *MYC*‐Δ1[1.3.1]TT, respectively. With a *T*_m_ of 81 °C, *MYC_del_*‐Δ1[1.1.1] shows the highest thermal stability of all quadruplexes, elevated by 6 and 11 °C compared to *MYC*‐Δ1,6 and *MYC*‐Δ1[1.3.1] with its longer two‐tetrad bridging central propeller loop. As anticipated, shorter loops enhance G4 stability also in the case of the snap‐back loop structure. However, with different enthalpic and entropic contributions to folding the considerable thermostability of the *MYC_del_*‐Δ1[1.1.1] quadruplex does not translate into the highest thermodynamic stability at ambient temperatures. In fact, *MYC*‐Δ1,6 features the most negative Δ*G* ^o^ of −7.7 and −5.3 kcal mol^−1^ among the G‐tract variants at 10 mm K^+^ and 293 and 310 K, respectively.

*MYC*‐Δ1[1.3.1] shows a more exothermic folding but a higher entropic penalty when compared to the *MYC_del_*‐Δ1[1.1.1] mutant with its 1‐nt central loop. Apparently, loop residues in this case may participate in additional hydrogen bond or stacking interactions, resulting in a more favorable enthalpic but unfavorable entropic contribution.[^29^, ^31^] Given negligible heat capacity effects (see above), the latter will generally result in a decreased thermal stability due to the strong temperature dependence of the *T*Δ*S* ^o^ term in the Gibbs‐Helmholtz equation.

Because the terminal 3’‐G fills an empty position to participate in the outer G‐tetrad of the snap‐back loop structure, its compatibility with a 3’‐extension seems critical for its formation within the nuclear hypersensitivity element IIII_1_. NMR structural studies have already shown that such an extended sequence preserves the same G4 topology with the 3’‐added nucleotide pointing away from the quadruplex G‐core.^[13]^ In line with the latter report, *MYC*‐Δ1[1.3.1]T shares a similar imino proton spectral region with *MYC*‐Δ1[1.3.1] and *MYC_del_*‐Δ1[1.1.1] albeit with small amounts of a minor species, indicating the formation of a major snap‐back loop quadruplex even within an extended sequence context (Figure 3). However, stabilities seem to be compromised with a Gibbs free energy of G4 folding declining to −2.9 kcal mol^−1^ at 310 K. Such destabilizing effects associated with an enhanced formation of other competitive species are even more pronounced for *MYC*‐Δ1[1.3.1]TT with a 2‐nucleotide 3’‐TT addition (Figure 3 and Table 2). Therefore, when solely based on thermodynamics these results raise doubts with respect to the relevance of such snap‐back loop structures under in vivo conditions.

### G‐register isomers

The two loop isomers *MYC*‐Δ1,6[1.2.1] and *MYC*‐Δ1,6[2.1.2] recruit the same four G‐tracts of *MYC* but incorporate different Gs of the two G_4_ tracts into their G‐quadruplex core. If blocking T substitutions are removed, a dynamic exchange between such G‐register isomers can occur through a sliding mechanism without complete refolding, entropically stabilizing the folded state.[^15^, ^19^] Only looking at the individual isomers, *MYC*‐Δ1,6[1.2.1] has a melting temperature higher by 13 °C as compared to *MYC*‐Δ1,6[2.1.2]. This is expected based on the longer loop lengths of the latter with their known destabilizing effects in parallel G4s. Also, a higher stability of *MYC*‐Δ1,6[1.2.1] is associated with a more favorable enthalpy of folding by about −11 kcal mol^−1^ that is only partially counteracted by a larger entropic penalty to give a ΔΔ*G*° of ≈−2.5 kcal mol^−1^ at 310 K. It should be noted that differences in Δ*H*° of −7 and −16 kcal mol^−1^ between *MYC*‐Δ1,6[1.2.1] and *MYC*‐Δ1,6[2.1.2] in buffer solutions containing 7.5 and 20 mm K^+^ have independently been determined previously based on a two‐state transition.[^17^, ^19^] This is in good agreement with the present data considering larger differences upon increasing K^+^ concentrations (see above and ref. [18]).

### Impact of overhang and loop sequences

As a result of using *MYC* sequences adopting well‐defined NMR‐derived quadruplex structures with appropriate G→T/A substitutions combined with efforts to add flanking residues for preventing G4 aggregation,^[32]^ mutual comparison of thermodynamic parameters among native and mutated sequences is often hampered by altered overhang and loop residues. Clearly, a negligible or only small impact of G mutations in trapped quadruplex structures constitutes a key assumption when assessing the thermodynamics of different G4 folds. Whereas moderate changes in quadruplex melting temperatures have been reported for T/A substitutions in loops of *MYC* sequences,^[18]^ only minor thermodynamic perturbations were suggested for thymidine and 2’‐deoxyinosine substitutions based on a global thermodynamic analysis.^[19]^ Indeed, due to *MYC*‐Δ1,6 predominantly folding into the *MYC*‐Δ1,6[1.2.1] topology with a central 2‐nt loop as shown by NMR (see above), the mostly identical thermodynamic parameters determined for folding of these two sequences confirm that the dual G→T mutations in *MYC*‐Δ1,6[1.2.1] have only a negligible impact on the thermodynamics of G4 formation (Table 2).

Based on these findings, significant differences in the thermodynamic profiles of *MYC*‐Δ1,6 and *MYC*‐Δ5,6 are unexpected. Both sequences feature four consecutive G‐tracts G_3_‐G_4_‐G_3_‐G_4_ for *MYC*‐Δ1,6 and G_4_‐G_3_‐G_4_‐G_3_ for *MYC*‐Δ5,6 separated by single T or A nucleotides. Consequently, formation of a most favored *MYC*‐Δ5,6[1.2.1] fold of similar stability to *MYC*‐Δ1,6[1.2.1] may be anticipated. Apparently, the different overhang and/or loop residues must exert more significant effects on G4 stability for these two G‐tract variants. To search for the origin of the different thermodynamic stabilities, we substituted *MYC*‐Δ1,6 in its overhang to form *MYC_TT_*‐Δ1,6 with TT flanking residues preceding and following the 5’ and 3’ G‐tract and to become a pseudo‐inverted *MYC*‐Δ5,6 sequence with A↔T exchange in the loops. Interestingly, these changes had no effect on the G4 melting temperature but lowered the exothermic heat of folding by nearly 9 kcal mol^−1^ counteracted by a smaller amount of entropy losses. Overall, the amount of Gibbs free energy decreased by 1 kcal mol^−1^ at 310 K. Such effects are easily rationalized by overhang sequences involved in specific interactions to act as stabilizing terminal caps of quadruplexes.[^10^, ^18^] However, a large part of the observed ΔΔ*G*°_310_ difference of 2.8 kcal mol^−1^ between *MYC*‐Δ1,6 and *MYC*‐Δ5,6 and of the accompanied decrease in melting temperature by 10 °C must be attributed to the different loop residues (T‐GA‐T versus A‐TG‐A when only assuming 1.2.1 loop isomers).^[18]^ These results emphasize that depending on the particular structural context overhang sequences and loop nucleotides may have more significant effects on the thermodynamics of folding. Also, only comparing melting temperatures may be misleading in not revealing significant changes in enthalpic and entropic contributions that determine relative stabilities at ambient temperatures.

## Conclusions

The *MYC* sequence with its multiple G‐tracts may fold into various quadruplexes with potential relevance under physiological conditions. Whereas melting temperatures are convenient parameters for probing their thermodynamic stability, *T*_m_ values are not directly linked to free enthalpies of G4 formation at a given temperature, requiring information on enthalpic and entropic contributions to the (un)folding event. Also, a thermodynamic characterization based on a van ’t Hoff analysis of melting profiles is strictly based on a two‐state transition. This limits an evaluation of folding processes proceeding through intermediates or to unresolved transitions of a sequence with different coexisting quadruplex folds. To overcome these restrictions, a rigorous thermodynamic analysis using microcalorimetry is required to allow for a model‐free extraction of parameters.

With more than four consecutive G‐tracts in the *MYC* sequence, those quadruplexes with a looped‐out internal tract are highly unfavorable unless specifically stabilized by potential interactions with ligands or proteins. On the other hand, two quadruplexes with a very similar sequence context, *MYC*‐Δ1,6 and *MYC*‐Δ5,6, considerably differ in their thermodynamic stability. *MYC*‐Δ1,6, proposed to fold into the physiologically most relevant G4 and also being the most stable G4 species, draws its energetic benefits to a large extent from its favorable loop composition when compared to *MYC*‐Δ5,6. Thus, recruiting excessive G‐tracts in case of oxidative lesions may circumvent deleterious effects on G‐quadruplex formation but is expected to yield a less stable alternative fold. Also, competition by a snap‐back loop quadruplex involving the 3’‐terminal GG tract VI must be met with caution. Although as stable as *MYC*‐Δ1,6 when truncated, an extended 3’‐end will allow, yet destabilize this particular topology.

The presented comprehensive thermodynamic profiling forms a solid basis for rationalizing stabilities of coexisting G4 structures that together with additional modulating interactions are expected to determine relevant populations within the cellular environment. Studying quadruplex structures and their relative populations in vivo remains a challenge but may be possible by the use of NMR in conjunction with stable isotope labeling. Also, detailed insights into the thermodynamics of different quadruplex folds will further contribute to our understanding of G4 stabilities in general and aid in the prediction of major folding topologies in sequences with multiple G‐runs.

## Experimental Section

### Materials and sample preparation

DNA oligonucleotides were purchased from *TIB MOLBIOL* (Berlin, Germany) and further purified by ethanol precipitation. Concentrations were determined spectrophotometrically by measuring absorbances in an H_2_O solution at 80 °C with molar extinction coefficients calculated by a nearest neighbor model.[^33^, ^34^] Prior to measurements, oligonucleotide solutions with concentrations as used for the subsequent experiments were annealed by heating to 85 °C for 5 min followed by slow cooling to room temperature and storage in a refrigerator overnight. For the experiments both a low‐salt buffer (10 mm potassium phosphate, pH 7) and a high‐salt buffer (20 mm potassium phosphate, 100 mm KCl, pH 7) was used.

### Circular dichroism (CD)

Spectra were acquired with a Jasco J‐810 spectropolarimeter equipped with a thermoelectrically controlled cell holder. Measurements were performed with 1‐cm quartz cuvettes at 293 K on 5 μm quadruplex in either a low‐salt or high‐salt buffer. Spectra were recorded with a bandwidth of 1 nm, a response time of 1–2 s, and a scanning speed of 50 nm min^−1^ and finally blank‐corrected.

### NMR spectroscopy

NMR spectra were acquired on a Bruker Avance 600 MHz spectrometer equipped with an inverse ^1^H/^13^C/^15^N/^19^F quadruple resonance cryoprobehead and *z*‐field gradients. Quadruplexes were dissolved in a low‐salt buffer with 10 mm potassium phosphate, pH 7.0. For solvent suppression on the samples in 90 % H_2_O/10 % D_2_O a WATERGATE with w5 element was employed. Data were processed using Topspin 4.0.6. Proton chemical shifts were referenced relative to TSP.

### Differential scanning calorimetry (DSC)

DSC measurements were performed on a VP‐DSC (Malvern Instruments, Great Britain) with 50 μm oligonucleotide in a 10 mm potassium phosphate buffer, pH 7, unless otherwise stated. Samples were heated from 20 to 100–110 °C with a scan rate of 0.5 °C min^−1^. Equilibrium conditions were confirmed by a single experiment with a scan rate of 0.25 °C min^−1^, yielding a thermogram superimposable on the thermogram obtained with twice the scan rate. A buffer versus buffer scan was subtracted from the sample scan and cubic baselines were constructed for each transition. Melting temperatures *T*_m_ and calorimetric enthalpies Δ*H* ^o^_cal_ corresponding to the maximum of the DSC peak and the area under the heat capacity versus temperature curve were obtained from the baseline‐corrected profiles. DSC curves were fitted with a non‐two‐state model as implemented in the DSC analysis software. Here, the temperature dependence of the ratio of unfolded and folded population as given by the shape of the DSC thermogram is described by the van ’t Hoff relationship with Δ*H* ^o^_vH_≠Δ*H* ^o^_cal_. Changes in heat capacity Δ*C*_p_° are too small for their reliable determination and were set to zero, consistent with negligible heat capacity effects upon quadruplex folding.^[21]^ Reported thermodynamic parameters are average values with corresponding standard deviations from at least three independent experiments.

## Conflict of interest

The authors declare no conflict of interest.

## Supporting information

As a service to our authors and readers, this journal provides supporting information supplied by the authors. Such materials are peer reviewed and may be re‐organized for online delivery, but are not copy‐edited or typeset. Technical support issues arising from supporting information (other than missing files) should be addressed to the authors.

Supplementary
