# Supplementary material for: A Thermodynamic Perspective on Potential G‐Quadruplex Structures as Silencer Elements in the MYC Promoter
Source: Chemistry. 2020 Nov 19;26(71):17242–51. doi: 10.1002/chem.202002985 (PMC7839732; doi:10.1002/chem.202002985)
Supplement: Supplementary file 1 — Supplementary [file CHEM-26-17242-s001.pdf]

Chemistry–A European Journal

Supporting Information

A Thermodynamic Perspective on Potential G-Quadruplex Structures as Silencer Elements in the MYC Promoter

Jagannath Jana and Klaus Weisz*[a]

Author Contributions

J.J. Formal analysis: Equal; Investigation: Lead; Methodology: Equal; Validation: Equal; Writing - Original Draft: Equal; Writing - Review & Editing: Equal
K.W. Conceptualization: Lead; Formal analysis: Equal; Funding acquisition: Lead; Methodology: Equal; Resources: Lead; Supervision: Lead; Validation: Equal; Writing - Original Draft: Equal; Writing - Review & Editing: Equal.

Supporting Information

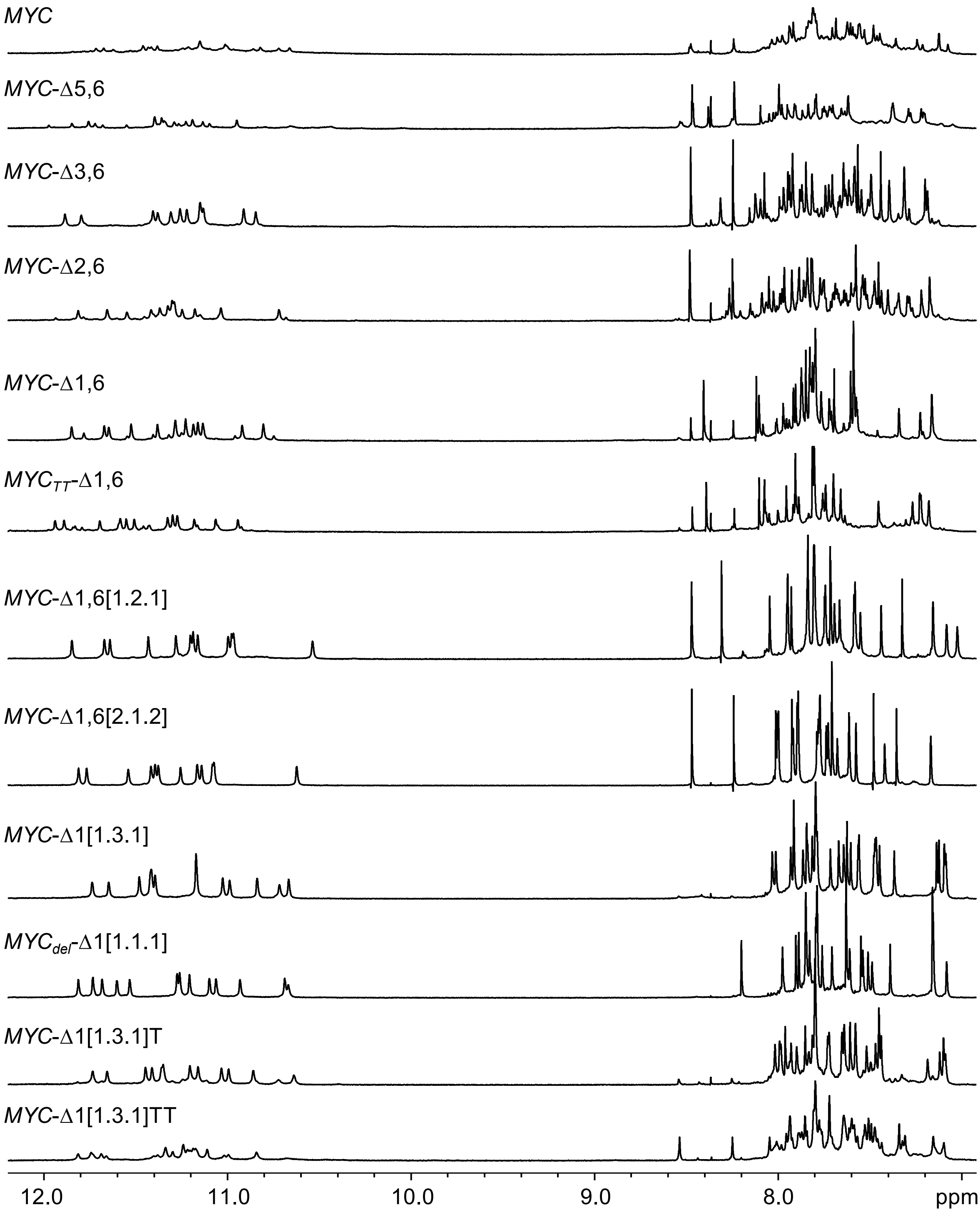

Figure S1. Imino and aromatic proton NMR spectral region of *MYC* sequence variants (0.2 mM) at 298 K in 10 mM potassium phosphate buffer, pH 7.

Gel electrophoresis

For non-denaturing polyacrylamide gel electrophoresis, annealed oligonucleotide samples (200 pmol in 10 μL per lane) were loaded onto a 15% polyacrylamide gel (acrylamide:bis-acrylamide 19:1). Separation was performed at room temperature in TBE buffer supplemented with 10mM KCl using a voltage of 80 V. Bands were visualized by staining with a 50 μM thiazole orange solution.

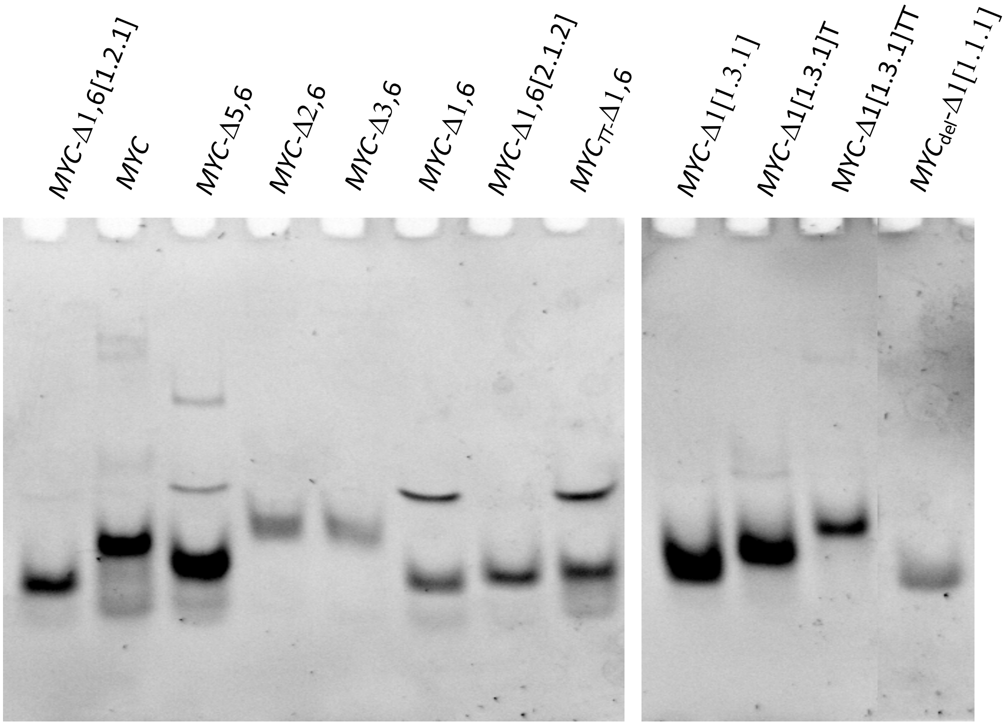

Figure S2. Non-denaturing polyacrylamide gel electrophoresis of *MYC* sequences after annealing in 10 mM potassium phosphate buffer, pH 7.

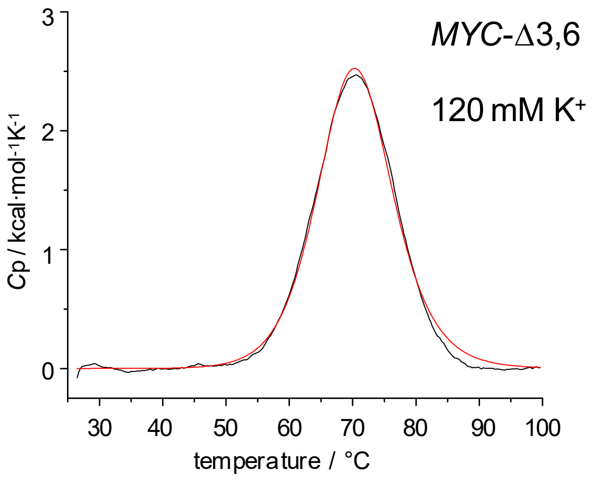

Figure S3. DSC thermogram for melting of *MYC*-Δ3,6 in 20 mM potassium phosphate, 100 mM KCl, pH 7. Fitted curve based on a non-two-state model with $\Delta H^{o}_{cal} \neq \Delta H^{o}_{vH}$ is shown in red.

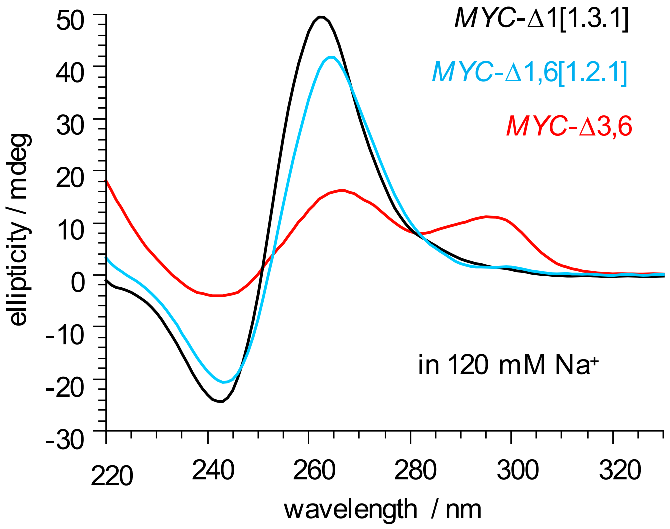

Figure S4. CD spectra of *MYC* sequence variants (5 µM) at 293 K in 20 mM sodium phosphate, 100 mM NaCl, pH 7.
